# Belimumab-induced remission in refractory lupus mesenteric vasculitis: a case report with 49-month follow-up

**DOI:** 10.3389/fimmu.2026.1687299

**Published:** 2026-03-04

**Authors:** Wen-jia Gao, Qi-feng Zang, Yan Xu, Yin-shan Zang

**Affiliations:** 1 Department of Rheumatology and Immunology, Jiangsu Province (Suqian) Hospital, Suqian, Jiangsu, China; 2 School of Life Sciences, Bengbu Medical University, Bengbu, Anhui, China

**Keywords:** belimumab, biologic agents, case report, glucocorticoid-sparing effect, mesenteric vasculitis, systemic lupus erythematosus

## Abstract

**Background:**

Lupus mesenteric vasculitis (LMV) is a rare but life-threatening gastrointestinal complication of systemic lupus erythematosus (SLE). Refractory cases pose significant therapeutic challenges due to limited treatment options and cumulative toxicity from long-term immunosuppressant therapy.

**Case presentation:**

A 46-year-old woman with SLE presented with recurrent abdominal pain, diarrhea, and malar rash over a 1-year period. Despite receiving standard immunosuppressive therapies—including glucocorticoids (GCs), cyclophosphamide, mycophenolate mofetil, and tacrolimus—she experienced multiple relapses of LMV between 2015 and 2020, confirmed by abdominal computed tomography showing bowel wall thickening and the characteristic “target sign.” In September 2020, belimumab (600 mg intravenous every 4 weeks) was initiated alongside a reduced-dose GC regimen. Over a 49-month treatment period (34 doses), the patient achieved sustained remission, with complete resolution of abdominal symptoms, normalization of computed tomography findings, stable Systemic Lupus Erythematosus Disease Activity Index scores (0–4), decreasing anti-double-stranded DNA titers (<20 IU/mL), and rising serum complement C3 levels (>0.6 g/L). GC dosage was successfully tapered to 2.5 mg/day without disease relapse.

**Conclusions:**

This case demonstrates belimumab’s efficacy in achieving the longest documented remission (49 months) in refractory LMV, highlighting its potential as a first-line biologic for steroid-dependent gastrointestinal vasculitis.

## Introduction

Systemic lupus erythematosus (SLE) is a chronic, multisystem autoimmune disorder that can affect virtually any organ. Lupus mesenteric vasculitis (LMV), which occurs in 0.2–9.7% of SLE patients (1), is a rare but potentially life-threatening gastrointestinal complication. LMV predominantly affects the small intestine, particularly the jejunum and ileum, leading to ischemia, infarction, and in severe cases, perforation (2, 3). The diagnosis is primarily based on clinical symptoms—such as abdominal pain and diarrhea—and imaging findings, including bowel wall thickening, the “target sign,” and mesenteric vessel engorgement on abdominal computed tomography (CT) scans (3).

First-line treatment for LMV typically involves high-dose glucocorticoids (GCs) combined with immunosuppressive agents (4). However, in refractory cases, relapses often occur during the tapering of GCs (5), leading to the need for prolonged high-dose immunosuppression, which can result in significant long-term toxicity.

Belimumab, a monoclonal antibody that inhibits B-lymphocyte stimulator (BLyS), has been approved for the treatment of active autoantibody-positive SLE (6). While its efficacy has been well-documented for renal and hematologic involvement in SLE (7), there are limited data on its use for the treatment of LMV. Here, we report a case of successful long-term remission in a patient with refractory LMV following treatment with belimumab.

## Case presentation

A 46-year-old female presented to our institution in February 2016 with a 1-year history of recurrent abdominal pain, diarrhea, and a malar rash, with acute symptom exacerbation in the preceding week. The patient initially developed abdominal pain, diarrhea, and vomiting in September 2015, which was misdiagnosed as acute appendicitis, leading to an appendectomy. However, her symptoms persisted postoperatively, followed by the emergence of a malar rash and alopecia. In 2016, a definitive diagnosis of SLE was established, confirmed by positive antinuclear antibodies (ANA+) and leukopenia (2.35 × 10^9^/L). Initial treatment consisted of intravenous (IV) methylprednisolone pulses and cyclophosphamide (cumulative dose: 2.4 g over 2 months), followed by oral prednisone, mycophenolate mofetil (MMF), and maintenance cyclophosphamide. From 2016 onward, hydroxychloroquine (0.2 g twice daily) was also consistently administered. The patient had no significant medical history. The patient denied a history of smoking or alcohol consumption. There was no family history of SLE or other genetic disorders.

From 2017 to 2020, the patient experienced multiple relapses of LMV, confirmed by abdominal CT imaging, necessitating repeated IV methylprednisolone pulses and adjustments in immunosuppressive therapy, including tacrolimus, re-initiation of cyclophosphamide, and tripterygium glycosides. Disease flares consistently occurred when prednisone was tapered to doses ≤7.5 mg/day. Following a recurrence of LMV in July 2020, belimumab therapy was initiated in September 2020.

**Figure 1:**
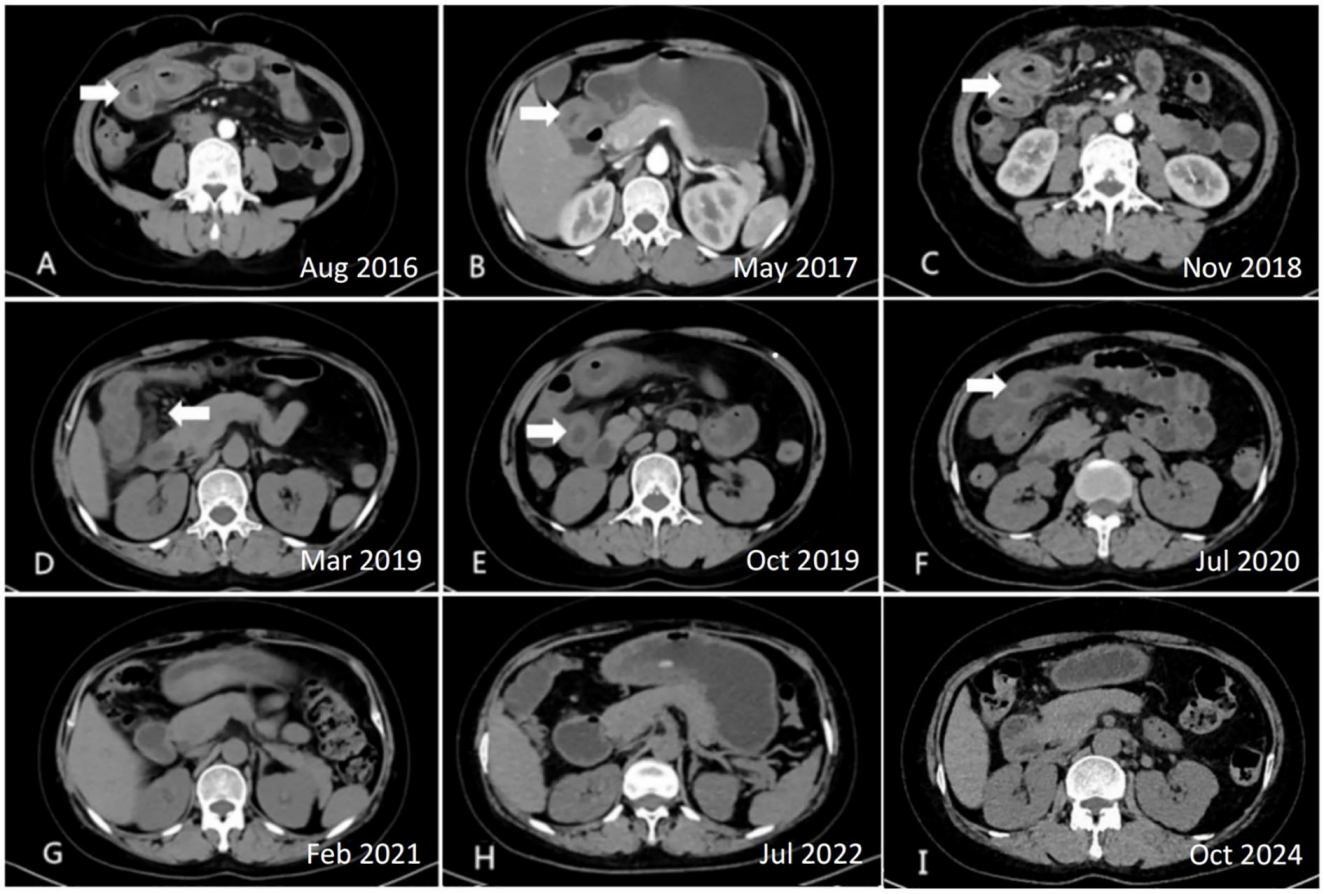
Serial abdominal computed tomography findings in lupus mesenteric vasculitis (LMV) before and after belimumab treatment. **(A-F)** Active LMV flares demonstrating bowel wall thickening,”target sign,” and “comb sign” (2016–2020); **(G-I)** Normalized bowel wall post-belimumab (2021–2024).

Upon admission, the patient was alert, hemodynamically stable, and had normal vital signs. Dermatological examination revealed a fixed, dark-red, butterfly-shaped rash across the nasal bridge and cheeks. Abdominal examination showed diffuse tenderness on deep palpation, but no rebound tenderness, guarding, or rigidity. Bowel sounds were normoactive. Musculoskeletal assessment revealed no joint swelling, synovitis, or tenderness. Initial serological testing revealed a positive ANA titer of 1:320 with a speckled immunofluorescence pattern. Anti-double-stranded DNA (anti-dsDNA) antibodies were significantly elevated to 46.16 IU/mL (reference range: <20 IU/mL). Additional autoantibody testing was positive for anti-Smith, anti-ribonucleoprotein, and anti-ribosomal P antibodies. Complement analysis showed marked hypocomplementemia, with C3 levels reduced to 0.46 g/L (normal range: 0.79–1.52 g/L) and C4 levels diminished to 0.08 g/L (normal range: 0.16–0.38 g/L). Hematological assessment revealed leukopenia (2.35 × 10^9^/L). Renal function testing identified proteinuria of 0.8 g/24h *via* quantitative urinalysis. Serial abdominal CT examinations performed during disease flares between 2016 and 2020 consistently demonstrated characteristic features of active mesenteric vasculitis. All six scans revealed diffuse circumferential bowel wall thickening (measuring 8–12 mm) with accompanying submucosal edema. The pathognomonic “target sign,” characterized by trilaminar wall enhancement with mucosal hyperenhancement, hypodense submucosal edema, and serosal enhancement, was evident in segments of the jejunum and ileum (**Figures 1A–F**). Additional findings included mesenteric fat stranding and engorgement of the vasa recta, presenting as the “comb sign” (**Figure 1D**).

The patient was diagnosed with SLE and LMV, accompanied by a Systemic Lupus Erythematosus Disease Activity Index (SLEDAI) score of 21, indicating severe disease activity. From 2015 to 2020, the patient underwent a treatment regimen involving repeated high-dose IV methylprednisolone pulses at 80 mg/day, supplemented with a combination of immunosuppressants, including cyclophosphamide (total cumulative dose: 8.8 g), MMF, tacrolimus, and tripterygium glycosides. Additionally, oral GCs (prednisone) were initiated at a dose of 30 mg/day; however, the patient was unable to maintain remission at doses below 7.5 mg/day.

In September 2020, the patient transitioned to a belimumab-based regimen, continuing until October 2024. This regimen involved IV belimumab administration at a dose of 600 mg, initially given 8 times every 4 weeks, followed by maintenance every 8 weeks, for a total of 34 doses. Concurrent therapies included prednisone (initiated at 15 mg/day, tapered by 2.5 mg every 4 weeks if SLEDAI ≤4 and no LMV symptoms, reaching 2.5 mg/day by Month 10 of belimumab therapy), MMF at 0.75 g daily, hydroxychloroquine at 0.2 g twice daily, and a brief continuation of cyclophosphamide (total cumulative dose: 4 g) before discontinuation. The treatment timeline is presented in **Table 1**.

**Table 1:** Longitudinal summary of clinical course, therapeutic interventions, and outcomes.

Time period	Clinical manifestations	Treatment regimen	Key outcomes
Sep 2015	Abdominal pain, diarrhea, vomiting	Misdiagnosed as appendicitis; appendectomy	Symptoms persisted postoperatively
2016	Malar rash, alopecia	IV methylprednisolone pulses + CYC (cumulative 2.4g); oral prednisone, HCQ,MMF	SLE diagnosis: ANA+, leukopenia (2.35×10^9^/L); relapse during GC taper
2017–2020	Recurrent LMV flares	Repeated IV GC pulses + IS agents (TAC, CYC, HCQ, tripterygium)	Relapses at prednisone ≤7.5 mg/day; CT-confirmed bowel wall thickening/”target sign”
Sep 2020	Acute LMV recurrence	Belimumab initiation (600 mg IV q4w) + prednisone 15 mg/day+MMF 0.75g/day+HCQ,	Rising anti-dsDNA (>20 IU/mL), low C3 (<0.6 g/L)
2020–2024	Asymptomatic remission	Belimumab (34 doses) + GC taper to 2.5 mg/day + MMF 0.75 g/day+HCQ 0.2g BID	Anti-dsDNA <20 IU/mL, C3 >0.6 g/L; SLEDAI 0–4; normalized CT

CYC, cyclophosphamide; MMF, mycophenolate mofetil; TAC, tacrolimus; HCQ, hydroxychloroquine.

**Figure 2:**
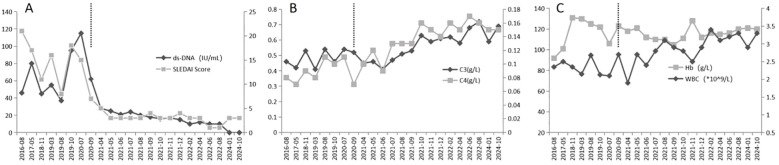
Longitudinal laboratory and disease activity parameters before and after belimumab treatment. **(A)** Anti-dsDNA titers and SLEDAI scores; **(B)** Complement C3 and C4 levels; **(C)** Hemoglobin and white blood cell counts. The vertical dashed line indicates the initiation of belimumab therapy in September 2020.

The patient was followed for 49 months, from September 2020 to October 2024. Clinically, the patient experienced complete resolution of abdominal pain and diarrhea. Laboratory results demonstrated consistently low anti-dsDNA levels (<20 IU/mL), normalization of complement component C3 (>0.6 g/L), and stabilization of the leukocyte count above 3.0 × 10^9^/L. Disease activity, as assessed by the SLEDAI score, remained consistently low, with scores ranging from 0 to 4 throughout the 49-month period (**Figures 2A–C**). Serial imaging studies, including CT scans, revealed complete resolution of previously observed bowel wall thickening and the target sign (**Figures 1G–I**). Furthermore, the patient successfully maintained a low dose of prednisone (2.5 mg/day) for over 24 months without experiencing any disease flare and remained in remission at the last follow-up in October 2024. Notably, no serious infections or adverse events related to belimumab use were reported during the follow-up period, indicating a favorable safety profile for this treatment regimen.

## Discussion

LMV is a severe gastrointestinal complication of SLE, with mortality rates as high as 11% when complicated by bowel infarction (2). In this case, we demonstrate the efficacy of belimumab in achieving sustained remission (49 months) and GC reduction in a patient with GC-dependent, immunosuppressant-refractory LMV, a condition in which conventional therapies failed over a span of six years. This outcome aligns with the pathogenesis of LMV: elevated BLyS promotes the survival of autoreactive B-cells and the deposition of immune complexes in the mesenteric vasculature, which triggers fibrinoid necrosis and thrombosis (8, 9). The patient’s serological profile further supports known risk factors for LMV (3), as these antibodies are implicated in endothelial activation and complement-mediated vascular injury (10).

Notably, histopathologic confirmation via intestinal biopsy was not pursued in this case. While biopsy remains the gold standard for vasculitis diagnosis, it carries significant risks in leukopenic patients (e.g., her leukopenia of 2.35×10^9^/L) and those on high-dose immunosuppressants, particularly with active intestinal ischemia (3). The characteristic CT ‘target sign’ combined with serologic SLE activity markers (hypocomplementemia, anti-dsDNA positivity) and clinical response to immunosuppression provided sufficient diagnostic certainty, aligning with current LMV management guidelines (1, 3).

The primary pathological feature of LMV is inflammatory ischemic vasculitis, underscoring the need for prompt initiation of aggressive anti-inflammatory and immunosuppressive therapy upon diagnosis. The typical therapeutic regimen involves high-dose intravenous GCs and cyclophosphamide, which is particularly crucial in cases of recurrent LMV (1). Our patient’s recurrent disease flares at prednisone doses ≤7.5 mg/day exemplify the limitations of this approach. Belimumab, by inhibiting BLyS, prevents the differentiation of B-cells into autoantibody-producing plasmablasts (11), directly targeting the underlying pathophysiology. A recent case report confirmed the efficacy of belimumab in treating a case of pan-gastrointestinal lupus (12). The observed serological normalization and resolution of CT “target signs” reflect the downstream effects of this targeted therapy.

Remarkably, belimumab enabled unprecedented GC reduction while maintaining disease remission. This GC-sparing effect is consistent with findings from multinational observational studies, which report a 43% reduction in average GC dosage within six months (13). However, the magnitude of this effect in refractory LMV is exceptional. The belimumab 10 mg/kg group demonstrated a greater reduction in GC dosage, with a higher proportion of participants reducing their dose by at least 50% compared to placebo. Multiple randomized controlled trials have confirmed that belimumab leads to significant improvements in GC dosage reduction (14). Our case extends these benefits to gastrointestinal vasculitis, a manifestation that has been underrepresented in belimumab trials.

While existing literature primarily documents the efficacy of belimumab in lupus nephritis and hematologic manifestations of SLE (6, 7), evidence specifically related to LMV, particularly refractory LMV, remains scarce. Our radiologically confirmed remission over 49 months provides compelling clinical evidence supporting its use in refractory LMV. Importantly, the concurrent use of mycophenolate mofetil may have enhanced efficacy by suppressing T-cell-dependent B-cell activation (15). This combination was maintained to leverage the potential synergistic effects, provide additional immunomodulatory cover during the critical glucocorticoid-tapering phase, and support long-term remission stability. Nonetheless, the pivotal therapeutic change is clearly attributed to belimumab, as evidenced by the temporal correlation between symptom resolution and its initiation.

This study has several limitations and therapeutic challenges that warrant consideration. The single-case design inherently limits generalizability, though the 49-month follow-up provides valuable long-term data requiring validation in multicenter cohorts. Histopathological confirmation was precluded by the patient’s leukopenic risk and active ischemia, highlighting the need for future endoscopic evaluations when feasible. While concomitant MMF use may confound therapeutic attribution, the temporal correlation of symptom resolution with belimumab initiation (post-MMF failure) strongly implicates its primary role. Broader challenges include the lack of validated LMV activity biomarkers, undefined biologic sequencing, and global disparities in biologic access. Mechanistically, serial BLyS or B-cell subset measurements were unavailable; incorporating these in future studies could identify predictive response markers.

## Conclusion

This case provides the longest documented follow-up (49 months) of belimumab-induced remission in refractory LMV. It demonstrates that targeted BLyS inhibition can achieve sustained gastrointestinal remission and unprecedented glucocorticoid reduction in conventionally treatment-resistant cases. These findings advocate for belimumab as a first-line biologic in refractory LMV and underscore the need for prospective trials focusing on SLE-related gastrointestinal vasculitis.

## Data Availability

The original contributions presented in the study are included in the article/supplementary material. Further inquiries can be directed to the corresponding author.
